# A Combination of GM-CSF and Released Factors from Gamma-Irradiated Tumor Cells Enhances the Differentiation of Macrophages from Bone Marrow Cells and Their Antigen-Presenting Function and Polarization to Type 1

**DOI:** 10.3390/medicines8070035

**Published:** 2021-07-04

**Authors:** Lichao Chen, Shoji Imamichi, Ying Tong, Yuka Sasaki, Takae Onodera, Satoshi Nakamura, Hiroshi Igaki, Jun Itami, Mitsuko Masutani

**Affiliations:** 1 Department of Molecular and Genomic Biomedicine, Nagasaki University Graduate School of Biomedical Sciences, Nagasaki 852-8523, Japan; bb55317801@ms.nagasaki-u.ac.jp (L.C.); simamich@ncc.go.jp (S.I.); bb55320022@ms.nagasaki-u.ac.jp (Y.T.); jj20210059@ms.nagasaki-u.ac.jp (Y.S.); takae-o@nagasaki-u.ac.jp (T.O.); 2 Radioisotope Division, Lab of Collaborative Research, Division of Cellular Signaling, National Cancer Center Research Institute, Tokyo 104-0045, Japan; 3 Division of BNCT, EPOC, National Cancer Center, Tokyo 104-0045, Japan; satonaka@ncc.go.jp (S.N.); hirigaki@ncc.go.jp (H.I.); jitami@ncc.go.jp (J.I.); 4 Department of Radiation Oncology, National Cancer Center Hospital, Tokyo 104-0045, Japan

**Keywords:** GM-CSF, macrophage, radiotherapy

## Abstract

Granulocyte-macrophage colony-stimulating factor (GM-CSF) promotes dendritic cell differentiation from precursors, and consequently, enhances the antigen presentation process and adaptive immune responses. With such functions, GM-CSF has been used as immunotherapy in combination with radiotherapy for cancer treatment to augment the survival and activity of immune cells. However, an immune-suppressive tumor microenvironment may cause anergy of T cells. It has also been reported that GM-CSF contributes to the development of myeloid-derived suppressor cells from the precursors. In this study, to analyze the combined effect of GM-CSF and released factors from cancer cells after gamma-ray irradiation on bone marrow cell differentiation and dynamics, we established an in vitro culture system using mouse bone marrow cells, GM-CSF, and conditioned medium from gamma ray irradiated mouse melanoma B16 cells at 24 Gy. We analyzed the gene expression changes of the bone marrow-derived cells on day 6. The results showed that GM-CSF dose-dependently enhanced the differentiation of macrophages from bone marrow cells, their antigen-presenting function and polarization to type I. The results implied the induced macrophages from the bone marrow may potentially contribute to tumor immune responses in a systemic manner when GM-CSF is boosted during photon-beam radiation therapy.

## 1. Introduction

Granulocyte-macrophage colony-stimulating factor (GM-CSF) is involved in the cell proliferation, differentiation, and survival of leukocytes [1,2,3]. Taking into account the fact that GM-CSF is able to activate and process the presentation of tumor-associated antigens by dendritic cell (DC)s, GM-CSF was studied in clinical trials by overexpressing in tumor cells, but significant therapeutic outcomes were not obtained [4,5]. As an immunoadjuvant, GM-CSF has been used during radiotherapy to increase immune cell activation and survival. Of note, around 30% of patients showed shrinkage of tumors at non-irradiated sites as an abscopal response, although the mechanism is not clearly understood [6]. The hypothesis for abscopal response induction mediated, at least partly by GM-CSF, is that GM-CSF systemically may promote the maturation of DCs. Consequently, the presentation of tumor antigen to naive T cells may be improved. The activated tumor antigen-specific T cells are then able to target the tumor cells at the irradiated and also non-irradiated metastasized sites [7]. However, it has been reported that GM-CSF treatment induced the differentiation of myeloid progenitors into myeloid-derived suppressor cells (MDSC), but not DCs in vitro and in vivo [8,9,10,11]. MDSC cells are immature myeloid cells and are precursors of DCs. They suppress the immune response and improve tumor growth [12,13]. On the other hand, GM-CSF stimulates the differentiation and activation of macrophages, which also function as antigen-presenting cells [14,15,16]. In a tumor microenvironment, tumor-associated macrophages form up to 50% of tumor mass and play important roles in tumor development [17,18]. Taken together, to understand the effect of GM-CSF on immune cells in tumor environment during radiotherapy, here we established an in vitro culture system. Immune cells, including DCs and macrophages, are recruited to tumor sites from blood, bone marrow, and lymph nodes. In this study, we cultured mouse bone marrow cells with recombinant mouse GM-CSF in a conditioned medium of mouse melanoma B16 cells collected 24 h after γ-irradiation. After 6 days of culture, adherent cells that were induced from the in vitro culture system were pan-macrophage marker F4/80 positive cells. The induced number of macrophages was higher when a conditioned medium from γ-irradiated B16 cells was used. The expression level of the genes, including *H2-Ab1*, *Cd86*, *Il12b*, and *Ccr7* [19,20,21,22] that promote the function of antigen presentation was increased dose-dependently with the concentration of GM-CSF after γ-irradiation. These results implied that macrophages are systemically induced from bone marrow cells in the combined action of GM-CSF and released factors from tumor cells and may affect tumor microenvironment and responses during radiotherapy.

## 2. Materials and Methods

### 2.1. Gamma Ray Irradiation

B16 cells were cultured in Dulbecco’s modified Eagle’s medium (12800-017, Gibco, Life Technologies Corp., Carlsbad, CA, USA) with 10% fetal bovine serum (FBS, 10270, Gibco, Life Technologies Corp., Carlsbad, CA, USA) and 1% penicillin and streptomycin (P/S, Gibco, Life Technologies Corp., Carlsbad, CA, USA). 9 × 10^5^ B16 cells in 5 mL medium were irradiated with 24 Gy of γ-ray using ^137^Cs γ-emitting irradiator (PS-3100SE, Pony Industry, Osaka, Japan) or mock-irradiated. The medium was harvested 24 h after irradiation as the conditioned medium (CM).

### 2.2. In Vitro Culture of Mouse Bone Marrow Cells

The animal studies were performed according to relevant national and international guidelines for animal welfare. All experiment protocols were approved by the Committee for Ethics in Animal Experimentation, and the experiments were conducted in accordance with the Guideline for Animal Experiments of the National Cancer Center (application number: T17-052; date of approval: 8 June 2017). Bone marrow cells were isolated from the femurs of C57BL/6 mice (Clea Japan, Inc., Tokyo, Japan), male, 10 weeks old. 4 × 10^5^ bone marrow cells were then cultured in 1.5 mL culture medium (RPMI 1640, 10% FBS, 1% P/S) and 0.5 mL of conditioned medium. Recombinant mouse GM-CSF (415-ML, RD systems, Minneapolis, MN, USA) was added at different concentrations. The medium was refreshed on day 3. After 6 days of culture, the loosely/non-adherent cells and adherent cells were counted and then harvested separately for further analysis.

### 2.3. Flow Cytometry

After 6 days of culture, the cells were harvested by cell scraper, and a single cell suspension was prepared in phosphate-buffered saline (PBS) containing 10% of FBS on ice. Fc blocking was performed using rat anti-mouse CD16/CD32 (553141, BD Pharmingen, Franklin Lakes, NJ, USA) for 20 min on ice [23]. After that, cell surface staining was performed by adding anti-mouse F4/80 (Brilliant Violet 605, 123133, Biolegend, San Diego, CA, USA) and incubated for 30 min on ice in the dark. The mouse lymph node cells were used as a positive control. After washing twice with PBS, the cells were then resuspended in ice-cold PBS with 10% FBS for the flow cytometric analysis using a BD LSRFortessa cell analyzer (BD Biosciences, San Jose, CA, USA). The mouse bone marrow cells and lymph node cells were kept in cell freeze media (Bambanker, NIPPON Genetics, Bunkyo-ku, Tokyo, Japan) and were stored at −80 °C before experiments.

### 2.4. Real Time PCR Analysis

The RNA was isolated from the harvested cells following the manufacturer’s instructions of Isogen (Nippongene, Tokyo, Japan). The cDNA was then synthesized from the RNA using the High-capacity cDNA Reverse Transcription Kit (4368814, Applied Biosystems, Waltham, MA, USA). Real-time PCR was performed by using the SYBR Select Master Mix (4472908, Applied Biosystems, Waltham, MA, USA) and a PCR instrument (StepOnePlus Real-time PCR System, Applied Biosystems, Waltham, MA, USA). The housekeeping gene *Actb* was used as the internal control. Gene-specific primers are shown in Table 1.

### 2.5. Statistical Analysis

Statistical analysis was performed using JMP Pro software (15.0.0, SAS Institute lnc., Cary, NC, USA, 2019) with the Wilcoxon rank-sum test method. Significance is denoted by asterisk. *, *p* < 0.05.

## 3. Results

### 3.1. Conditioned Medium from Gamma-Irradiated B16 Cells Plus GM-CSF Enhanced the Differentiation of Macrophages from Bone Marrow Cells

**Figure 1:**
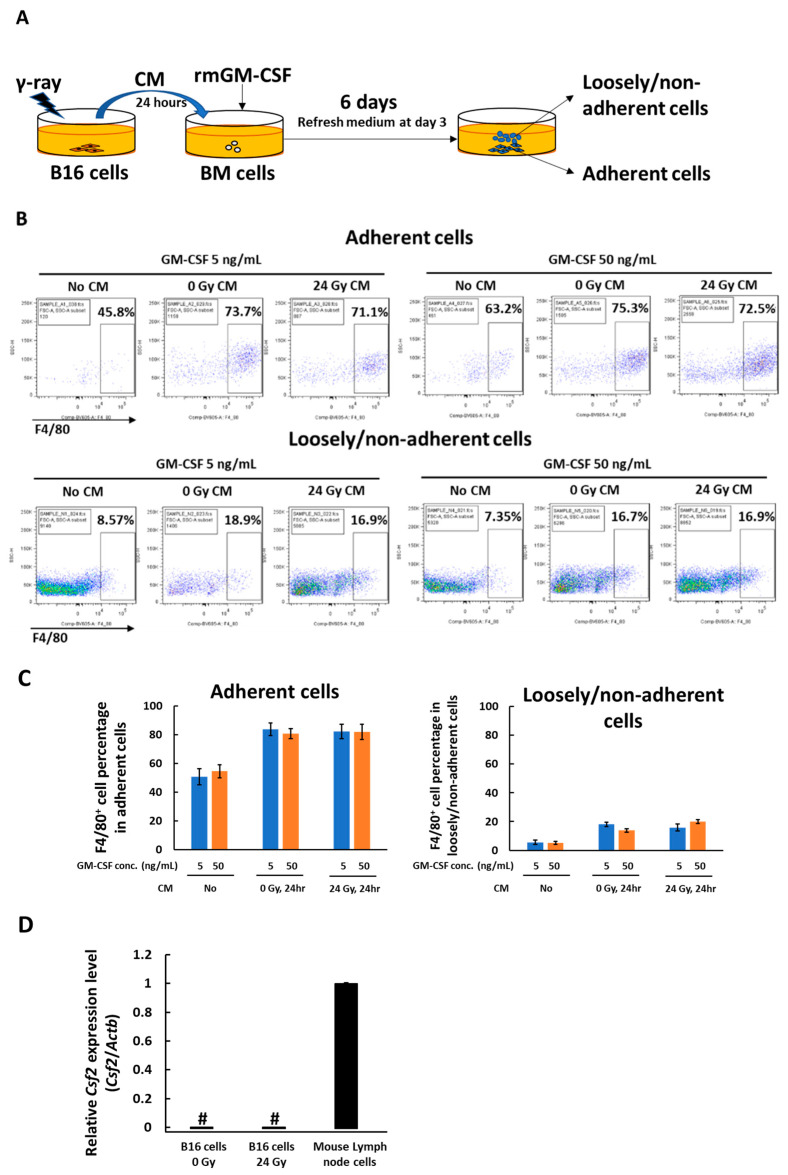
In vitro culture system for bone marrow cell differentiation in the presence or absence of GM-CSF and CM from non-irradiated or 24 Gy γ-irradiated B16 cells. (**A**) schema of the in vitro culture. (**B**) The adherent and loosely/non-adherent cells were recovered after culture for 6 days in the presence or absence of GM-CSF plus CM obtained from non-irradiated or 24 h after 24 Gy γ-irradiated B16 cells, stained with a mouse macrophage marker F4/80 antibody and analyzed by flow cytometry. (**C**) F4/80 positive cell percentages in adherent cells and loosely/non-adherent cells. (**D**) Real-time PCR analysis of *Csf2* gene expression in B16 cells 24 h after γ-irradiation. #, undetected; Mean ± SE, *n* = 3.

The femur bone-marrow cells from C57BL/6 mice were cultured in vitro with conditioned medium and recombinant GM-CSF (Figure 1A). After 6 days of culture, the loosely/non-adherent cells and adherent cells were harvested and stained with a mouse pan-macrophage marker F4/80 antibody. There was almost no adherent or loosely/non-adherent cell survival in the absence of GM-CSF. On the other hand, in the presence of GM-CSF, adherent cells loosely/non-adherent cell were survived on day 6. Flow cytometry analysis results showed the majority of adherent cells were positively stained with F4/80 antibody, indicating that macrophage differentiation was induced, while the loosely/non-adherent cells contained less than 20% positivity to F4/80 antibody staining (Figure 1B,C). We checked the expression of the *Csf2* gene that encodes GM-CSF in B16 cells before and after γ-irradiation (Figure 1D). Mouse lymph node cells were used as a positive control. B16 cells did not express *Csf2* before or after γ-irradiation. This indicates that the effect of GM-CSF level could be examined in this system by changing the amount of added recombinant GM-CSF.

**Figure 2:**
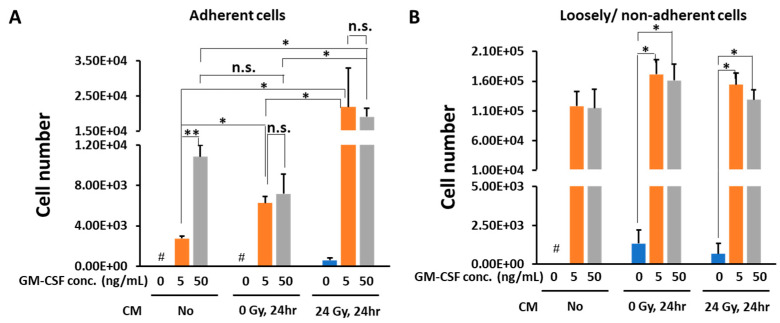
Cell number changes of bone marrow cells on day 6 after culture with GM-CSF and CM from non-irradiated or 24 h after 24 Gy γ-irradiated B16 cells. The cell number counted results of the adherent cells (**A**) and loosely/non-adherent cells (**B**). Mean ± SE, *n* = 3. *, *p* < 0.05. **, *p* < 0.005. #, no living cells.

Addition of GM-CSF induced macrophages dose-dependently, even in the absence of CM. There was no significant difference in macrophage cell number between different concentrations of GM-CSF when the cells were cultured in the presence of CM obtained from non-irradiated mouse melanoma B16 cells. Notably, CM obtained 24 h after 24 Gy γ-irradiated B16 culture induced macrophages approximately 3-fold more compared with CM from non-irradiated culture (Figure 2A). We also noted that CM from 24 Gy irradiated B16 cells in the absence of GM-CSF could induce low amounts of adherent cells.

Loosely/non-adherent cell numbers after 6 days of culture were higher than those of adherent cells. However, no dose-dependency in the cell number was observed between different GM-CSF concentrations in the absence of CM. Furthermore, there was no difference in the cell numbers in the presence of non-irradiated or the irradiated CM from B16 cells (Figure 2B).

Taken together, GM-CSF induced macrophage differentiation from bone marrow cells in the absence of B16 CM in vitro. The CM from 24 Gy irradiated B16 cells enhanced macrophage differentiation from bone marrow cells in the presence of GM-CSF, whereas loosely/non-adherent cell numbers were not significantly affected by the presence of CM. In this study, we focused on the properties of induced macrophages in the adherent cell population derived from bone marrow cells.

### 3.2. In the Presence of CM from 24 Gy Irradiated B16 Cells, the Antigen Presenting Function of Macrophages Increases Depending on GM-CSF Concentration

**Figure 3:**
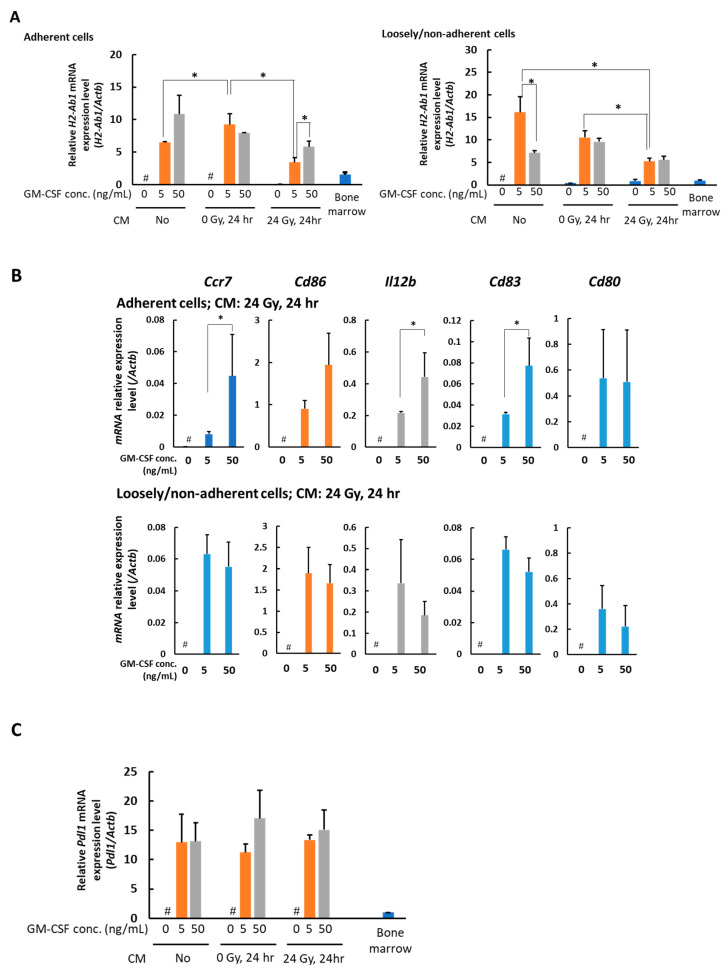
Real-time PCR analysis of the expression level of the genes involved in antigen presentation in the adherent and loosely/non-adherent cells. (**A**) *H2-Ab1* mRNA expression level. (**B**) *Ccr7*, *Cd80*, *Cd83*, *Cd86*, and *Il12b* mRNA expression levels. (**C**) *Pdl1* mRNA expression level in the adherent cells. Bone marrow cells on day 6 after culture with different concentrations of GM-CSF and CM obtained from non-irradiated or 24 h after 24 Gy γ-irradiated B16 cells. Mean ± SE, *n* = 3. *, *p* < 0.05. #, no living cells.

To further examine the phenotype of the macrophages, we analyzed the expression of several genes involved in the antigen-presenting function, including *H2-Ab1*, *Ccr7*, *Cd80*, *Cd83*, *Cd86*, *and Il12b*, using real-time PCR. *H2-Ab1* (encoding MHC II), as a marker for classic antigen present cells, was first tested. The data showed that the expression of MHCII in macrophages was suppressed in the presence of CM of 24 Gy irradiated B16 cells. Interestingly, this suppression was slightly recovered by the presence of increased concentrations of GM-CSF (Figure 3A). However, these types of changes were not observed in the loosely/non-adherent cells. The expression of *H2-Ab1* was decreased in the loosely/non-adherent cells when 0 or 24 Gy irradiated B16 CM was present (Figure 3A). The expression of the molecules that participate in the antigen presentation process*—Ccr7*, *Cd80*, *Cd83*, *Cd86*, and *Il12b*—were analyzed. In the adherent cells, the expression of genes of *Ccr7*, *Cd83*, and *Il12b*, but not *Cd80*, was augmented dose-dependently with GM-CSF concentration when the CM of 24 Gy irradiated B16 cells were present. An increasing tendency of gene *Cd86* expression levels was observed, depending on GM-CSF concentration for adherent cells, although there was no statistical significance (Figure 3B). In contrast, the levels of *Ccr7*, *Cd83*, *Cd86*, and *Il12b* were not dependent on GM-CSF concentration in loosely/non-adherent cells when the CM of 24 Gy irradiated B16 cells was present for 6 days (Figure 3B). The expression level of gene *Cd80* was not altered depending on GM-CSF concentration either in adherent cells or loosely/non-adherent cells (Figure 3B). *Pdl1* gene expression in the adherent cells was checked and no significant difference was observed with different conditions (Figure 3C).

**Figure 4:**
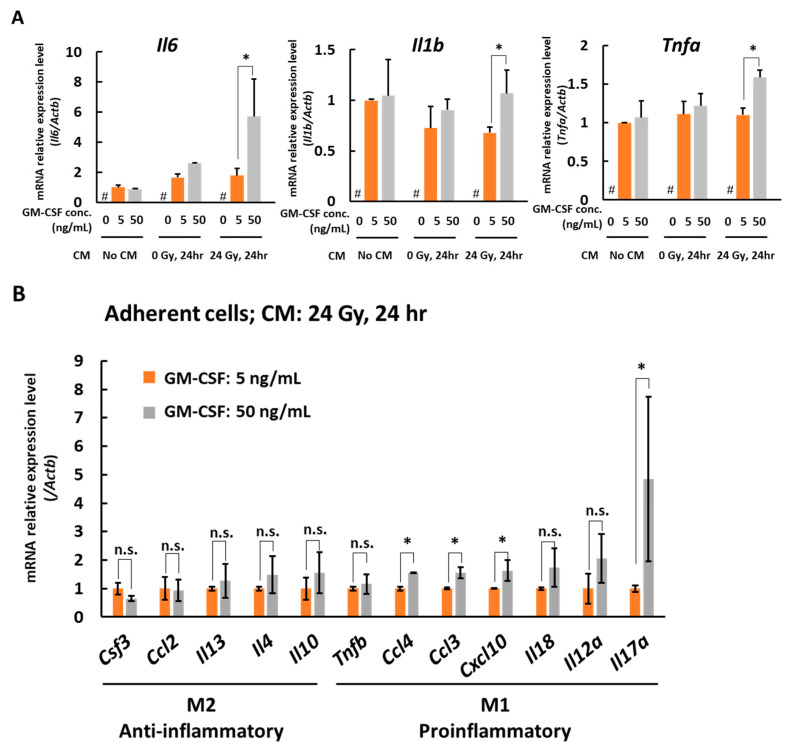
Real-time PCR analysis of the expression of type 1 macrophage markers and cytokine genes expression profiles in adherent cell fractions. (**A**) mRNA expression level of macrophage type 1 markers, *Il6*, *Il1b*, and *Tnfa*. Bone marrow cells on day 6 after culture with different concentrations of GM-CSF and CM obtained from non-irradiated or 24 h after 24 Gy γ-irradiated B16 cells. Mean ± SE, *n* = 3. *, *p* < 0.05. #, no living cells. (**B**) The cytokine gene expression profiles related to type 1 and type 2 macrophages. Adherent cells obtained from bone marrow cells on day 6 after culture with different concentrations of GM-CSF and CM obtained 24 h after 24 Gy γ-irradiated B16 cells. Mean ± SE, *n* = 3. *, *p* < 0.05. n.s., no significant difference.

We also analyzed the gene markers of type 1 macrophages, *Il6*, *Il1b*, and *Tnfa*. In adherent cells, the gene expression of *Il6*, *Il1b*, and *Tnfa* dose-dependently increased with the concentration of GM-CSF when the cells were cultured with CM of 24 Gy irradiated B16 cells for 6 days (Figure 4A). To further examine the macrophage properties, we analyzed cytokine and other macrophage-related factor expression profiles by real-time PCR. As shown in Figure 4B, anti-inflammatory macrophage cytokine genes, *Csf3*, *Ccl2*, *Il13*, *Il4*, and *Il10* [24,25,26] did not show changes with a statistically significant difference, whereas the expression of proinflammatory cytokine genes, *Ccl3*, *Ccl4*, *Cxcl10*, and *Il17a* [24,27,28,29], showed a dose-dependent increase with the concentration of GM-CSF when the bone marrow cells were cultured with the CM of 24 Gy irradiated B16 cells for 6 days, suggesting polarization to type I in this condition. Taken together, when the CM of 24 Gy irradiated B16 cells is present, GM-CSF is suggested to dose-dependently enhance the antigen-presenting function of the macrophages and may induce the macrophage polarization to type 1.

## 4. Discussion

GM-CSF has been used in tumor therapy not only for the enhancement of hematopoietic cells but also for its potential function to activate antigen-presenting cells, which can consequently activate tumor antigen-specific T cells to kill tumor cells. However, inconsistent clinical outcomes have been reported [30]. The complexity of the tumor microenvironment, including MDSCs, Treg cells, tumor-associated macrophages, and immunosuppressive cytokines, may have caused the low clinical anti-tumor responses [4,31]. The immunosuppressive condition may cause the anergy of tumor-specific T cells that can be induced by GM-CSF [32]. Furthermore, in the tumor microenvironment, GM-CSF is suggested to contribute to the development of MDSCs, which suppress T cell function. On the other hand, there are clinical data showing a 30% response of abscopal effect with treatment by x-ray radiotherapy/chemotherapy combined with GM-CSF administration, indicating a possibility of functional enhancement of antigen-presenting cells and systemic anti-tumor responses after irradiation. In this study, therefore, we established an in vitro culture system to analyze a combination effect of GM-CSF and γ-irradiation on mouse bone marrow cells. We used mouse melanoma B16 cells as tumor cells and C57BL/6J mouse-derived bone marrow cells. B16 cells were used herein because B16 cells lack *Csf2* gene expression before or after irradiation at 24 h, and we could examine the effect of GM-CSF level by changing the amount of added recombinant GM-CSF.

The gene expression profile data of macrophage-related factors and cytokines suggested that released factors from γ-irradiated tumor cells in combination with GM-CSF enhanced the development of type I macrophages from bone marrow cells and improved the antigen-presenting function of the macrophages, but not the DCs. The results implied that the induced type I macrophages may systemically affect the tumor microenvironment.

We only used the mouse melanoma B16 cells as the tumor cells for the source of conditioned medium, but the tumors of various types are expected to exert different effects on the differentiation of macrophages and DCs after γ-irradiation. Further analysis of these induced macrophages and DCs with flow cytometry and other methods should be able to delineate their classifications and phenotypes. Detailed antigen presentation assay may also be useful to analyze the antigen-presenting function of the induced macrophages.

The responsible released factors in the CM have not been characterized. The damage-associated molecular patterns (DAMPs), including high mobility group box protein 1 (HMGB1) that are known to be released from the γ-irradiation-induced damaged or dying cells may contribute to induce the macrophage differentiation [33]. Further analysis of the factors in the CM of tumor cells should be performed to identify and explore the underlying mechanism.

In this study, we used the CM of mouse melanoma cells irradiated at 24 Gy, which is the therapeutic dose used in particular photon-beam radiotherapies, such as stereotactic radiosurgery using LINAC and gamma-knife. Similar ranges of equivalent radiation doses are also applied for particle beam therapy, such as boron neutron capture therapy. Further analysis of irradiation dose-dependency study and time-course analysis for CM and in vivo analysis with xenograft tumors and with the administration of GM-CSF will be useful for the analysis of the systemic and local combinational effects of GM-CSF and radiation on local and systemic effects, including tumor growth and abscopal effects. This in vitro culture method may be useful to analyze the combinational effect of GM-CSF and released factors from γ-irradiated tumor cells on human bone marrow.

## 5. Conclusions

In conclusion, using an in vitro culture method for the assessment of mouse bone-marrow cell differentiation during tumor radiotherapy conditions, we demonstrated that a combination of GM-CSF and released factors from γ-irradiated tumor cells enhances the differentiation of macrophages from bone marrow cells and improves their antigen presenting function and polarization to type 1.

## Figures and Tables

**Table 1:** Gene specific primers.

Gene	Forward	Reverse
*Actb*	GCCAACCGTGAAAAGATGACC	GCGTGAGGGAGAGCATAGC
*Ccr7*	TCATTGCCGTGGTGGTAGTCTTCA	ATGTTGAGCTGCTTGCTGGTTTCG
*Cd83*	GTGGCACTGAGAGTGTGGAG	TTGGATCGTCAGGGAATAGG
*Cd80*	CTGGGAAAAACCCCCAGAAG	TGACAACGATGACGACGACTG
*Cd86*	ATCAAGGACATGGGCTCGTA	GAAGTTGGCGATCACTGACA
*H2-Ab1*	AGCCCCATCACTGTGGAGT	GATGCCGCTCAACATCTTGC
*Il12b*	ATGGAGTCATAGGCTCTGGAAA	CCGGAGTAATTTGGTGCTTCAC
*Il6*	CTGCAAGAGACTTCCATCCAG	AGTGGTATAGACAGGTCTGTTGG
*Il1b*	TGAAATGCCACCTTTTGACAG	CCACAGCCACAATGAGTGATAC
*Tnfa*	GGCAGGTCTACTTTGGAGTCAT	CAGAGTAAAGGGGTCAGAGTGG
*Csf3*	GCCACCTACAAGCTGTGTCACC	GCTGGCTTAGGCACTGTGTCTG
*Ccl2*	ATTGGGATCATCTTGCTGGT	CCTGCTGTTCACAGTTGCC
*Il13*	CCTGGCTCTTGCTTGCCTT	GGTCTTGTGTGATGTTGCTCA
*Il4*	GGTCTCAACCCCCAGCTAGT	GCCGATGATCTCTCTCAAGTGAT
*Il10*	TAACTGCACCCACTTCCCAG	AGGCTTGGCAACCCAAGTAA
*Tnfb*	TCACCTCAGACAGGACCCAT	AGCAGTGGCTGGCTTTTAGA
*Ccl4*	ATGAAGCTCTGCGTGTCTGC	CTGCCGGGAGGTGTAAGAGA
*Ccl3*	ACCATGACACTCTGCAACCAAGTC	GCGTGGAATCTTCCGGCTGTAG
*Cxcl10*	AGAGACATCCCGAGCCAACC	AGTCCCACTCAGACCCAGCAG
*Il18*	ATGCTTTCTGGACTCCTGCC	ATTGTTCCTGGGCCAAGAGG
*Il12a*	CCATCAACGCAGCACTTCAG	TCACCCTGTTGATGGTCACG
*Il17a*	TCTCTGATGCTGTTGCTGCT	CGTGGAACGGTTGAGGTAGT

## Data Availability

Not applicable.

## Abbreviations

Actb	actin beta
Ccr7	C-C motif chemokine receptor 7
Cd83	CD83 molecule
Cd80	CD80 molecule
Cd86	CD86 molecule
H2-Ab1	histocompatibility 2, class II antigen A, beta 1
Il12b	interleukin 12B
Il6	interleukin 6
Il1b	interleukin 1 beta
Tnfa	tumor necrosis factor a
Csf3	colony stimulating factor 3
Ccl2	C-C motif chemokine ligand 2
Il13	interleukin 13
Il4	interleukin 4
Il10	interleukin 10
Tnfb	tumor necrosis factor b
Ccl4	C-C motif chemokine ligand 4
Ccl3	C-C motif chemokine ligand 3
Cxcl10	C-X-C motif chemokine ligand 10
Il18	interleukin 18
Il12a	interleukin 12A
Il17a	interleukin 17A

## References

[1] Becher B., Tugues S., Greter M. (2016). GM-CSF: From Growth Factor to Central Mediator of Tissue Inflammation. Immunity.

[2] Avalos B.R. (1996). Molecular analysis of the granulocyte colony-stimulating factor receptor. Blood.

[3] Burgess A.W., Metcalf D. (1980). The nature and action of granulocyte-macrophage colony stimulating factors. Blood.

[4] Lawson D.H., Lee S., Zhao F., Tarhini A.A., Margolin K., Ernstoff M.S., Atkins M.B., Cohen G.I., Whiteside T.L., Butterfield L.H. (2015). Randomized, Placebo-Controlled, Phase III Trial of Yeast-Derived Granulocyte-Macrophage Colony-Stimulating Factor (GM-CSF) Versus Peptide Vaccination Versus GM-CSF Plus Peptide Vaccination Versus Placebo in Patients With No Evidence of Disease After Complete Surgical Resection of Locally Advanced and/or Stage IV Melanoma: A Trial of the Eastern Cooperative Oncology Group-American College of Radiology Imaging Network Cancer Research Group (E4697). J. Clin. Oncol..

[5] Simons J.W., Jaffee E.M., Weber C.E., Levitsky H.I., Nelson W.G., Carducci M.A., Lazenby A.J., Cohen L.K., Finn C.C., Clift S.M. (1997). Bioactivity of autologous irradiated renal cell carcinoma vaccines generated by ex vivo granulocyte-macrophage colony-stimulating factor gene transfer. Cancer Res..

[6] Golden E.B., Chhabra A., Chachoua A., Adams S., Donach M., Fenton-Kerimian M., Friedman K., Ponzo F., Babb J., Goldberg J. (2015). Local radiotherapy and granulocyte-macrophage colony-stimulating factor to generate abscopal responses in patients with metastatic solid tumours: A proof-of-principle trial. Lancet Oncol..

[7] Grass G.D., Krishna N., Kim S. (2016). The immune mechanisms of abscopal effect in radiation therapy. Curr. Probl. Cancer.

[8] Gargett T., Christo S.N., Hercus T.R., Abbas M., Singhal N., Lopez A.F., Brown M.P. (2016). GM-CSF signalling blockade and chemotherapeutic agents act in concert to inhibit the function of myeloid-derived suppressor cells in vitro. Clin. Transl. Immunol..

[9] Erlich Z., Shlomovitz I., Edry-Botzer L., Cohen H., Frank D., Wang H., Lew A.M., Lawlor K.E., Zhan Y., Vince J.E. (2019). Macrophages, rather than DCs, are responsible for inflammasome activity in the GM-CSF BMDC model. Nat. Immunol..

[10] Umansky V., Blattner C., Gebhardt C., Utikal J. (2016). The Role of Myeloid-Derived Suppressor Cells (MDSC) in Cancer Progression. Vaccines.

[11] Hutchison S., Sahay B., de Mello S.C., Sayour E., Lejeune A., Szivek A., Livaccari A., Fox-Alvarez S., Salute M., Powers L. (2019). Characterization of myeloid-derived suppressor cells and cytokines GM-CSF, IL-10 and MCP-1 in dogs with malignant melanoma receiving a GD3-based immunotherapy. Veter Immunol. Immunopathol..

[12] Veglia F., Perego M., Gabrilovich D. (2018). Myeloid-derived suppressor cells coming of age. Nat. Immunol..

[13] Umansky V., Sevko A., Gebhardt C., Utikal J. (2014). Myeloid-derived suppressor cells in malignant melanoma. J. Dtsch. Dermatol. Ges..

[14] Unanue E.R. (1984). Antigen-presenting function of the macrophage. Annu. Rev. Immunol..

[15] Rimaniol A.-C., Gras G., Verdier F., Capel F., Grigoriev V.B., Porcheray F., Sauzeat E., Fournier J.-G., Clayette P., Siegrist C.-A. (2004). Aluminum hydroxide adjuvant induces macrophage differentiation towards a specialized antigen-presenting cell type. Vaccine.

[16] Gordon S. (2007). The macrophage: Past, present and future. Eur. J. Immunol..

[17] Azizi E., Carr A.J., Plitas G., Cornish A.E., Konopacki C., Prabhakaran S., Nainys J., Wu K., Kiseliovas V., Setty M. (2018). Single-Cell Map of Diverse Immune Phenotypes in the Breast Tumor Microenvironment. Cell.

[18] Mantovani A., Marchesi F., Malesci A., Laghi L., Allavena P. (2017). Tumour-associated macrophages as treatment targets in oncology. Nat. Rev. Clin. Oncol..

[19] Unanue E.R., Turk V., Neefjes J. (2016). Variations in MHC Class II Antigen Processing and Presentation in Health and Disease. Annu. Rev. Immunol..

[20] Förster R., Davalos-Misslitz A.C., Rot A. (2008). CCR7 and its ligands: Balancing immunity and tolerance. Nat. Rev. Immunol..

[21] Schweitzer A.N., Borriello F., Wong R.C., Abbas A.K., Sharpe A.H. (1997). Role of costimulators in T cell differentiation: Studies using antigen-presenting cells lacking expression of CD80 or CD86. J. Immunol..

[22] Abdi K. (2002). IL-12: The role of p40 versus p75. Scand. J. Immunol..

[23] Andersen M.N., Al-Karradi S.N.H., Kragstrup T.W., Hokland M. (2016). Elimination of erroneous results in flow cytometry caused by antibody binding to Fc receptors on human monocytes and macrophages. Cytom. Part A.

[24] Sierra-Filardi E., Nieto C., Domínguez-Soto Á., Barroso R., Sánchez-Mateos P., Puig-Kroger A., López-Bravo M., Joven J., Ardavín C., Fernandez J.L.R. (2014). CCL2 shapes macrophage polarization by GM-CSF and M-CSF: Identification of CCL2/CCR2-dependent gene expression profile. J. Immunol..

[25] Wen Q., Kong Y., Zhao H.-Y., Zhang Y.-Y., Han T.-T., Wang Y., Xu L.-P., Zhang X.-H., Huang X.-J. (2019). G-CSF-induced macrophage polarization and mobilization may prevent acute graft-versus-host disease after allogeneic hematopoietic stem cell transplantation. Bone Marrow Transplant..

[26] Orihuela R., McPherson C.A., Harry G.J. (2016). Microglial M1/M2 polarization and metabolic states. Br. J. Pharmacol..

[27] Xuan W., Qu Q., Zheng B., Xiong S., Fan G.-H. (2014). The chemotaxis of M1 and M2 macrophages is regulated by different chemokines. J. Leukoc. Biol..

[28] Climaco-Arvizu S., Domínguez-Acosta O., Cabañas-Cortés M.A., Rodríguez-Sosa M., Gonzalez F.J., Vega L., Elizondo G. (2016). Aryl hydrocarbon receptor influences nitric oxide and arginine production and alters M1/M2 macrophage polarization. Life Sci..

[29] Chen J., Liao M.-Y., Gao X.-L., Zhong Q., Tang T.-T., Yu X., Liao Y.-H., Cheng X. (2013). IL-17A induces pro-inflammatory cytokines production in macrophages via MAPKinases, NF-κB and AP-1. Cell. Physiol. Biochem..

[30] Kaufman H.L., E Ruby C., Hughes T., Slingluff C.L. (2014). Current status of granulocyte–macrophage colony-stimulating factor in the immunotherapy of melanoma. J. Immunother. Cancer.

[31] Kirkwood J.M., Lee S., Moschos S.J., Albertini M.R., Michalak J.C., Sander C., Whiteside T., Butterfield L.H., Weiner L. (2009). Immunogenicity and antitumor effects of vaccination with peptide vaccine+/− granulocyte-monocyte colony-stimulating factor and/or IFN-α2b in advanced metastatic melanoma: Eastern Cooperative Oncology Group Phase II Trial E1696. Clin. Cancer Res..

[32] Gajewski T.F., Schreiber H., Fu Y.-X. (2013). Innate and adaptive immune cells in the tumor microenvironment. Nat. Immunol..

[33] Krysko D., Garg A.D., Kaczmarek A., Krysko O., Agostinis P., Vandenabeele P. (2012). Immunogenic cell death and DAMPs in cancer therapy. Nat. Rev. Cancer.

